# Information Entropy Analysis of a PIV Image Based on Wavelet Decomposition and Reconstruction

**DOI:** 10.3390/e26070573

**Published:** 2024-06-30

**Authors:** Zhiwu Ke, Wei Zheng, Xiaoyu Wang, Mei Lin

**Affiliations:** 1 Science and Technology on Thermal Energy and Power Laboratory, Wuhan Second Ship Design and Research Institute, Wuhan 430025, China; zhengwei@mail.ustc.edu.cn; 2 School of Energy and Power Engineering, Xi’an Jiaotong University, Xi’an 710049, China; tongli97@stu.xjtu.edu.cn (X.W.); janeylinm@mail.xjtu.edu.cn (M.L.)

**Keywords:** particle image velocimetry, image processing, wavelet decomposition and reconstruction, information entropy

## Abstract

In particle image velocimetry (PIV) experiments, background noise inevitably exists in the particle images when a particle image is being captured or transmitted, which blurs the particle image, reduces the information entropy of the image, and finally makes the obtained flow field inaccurate. Taking a low-quality original particle image as the research object in this research, a frequency domain processing method based on wavelet decomposition and reconstruction was applied to perform particle image pre-processing. Information entropy analysis was used to evaluate the effect of image processing. The results showed that useful high-frequency particle information representing particle image details in the original particle image was effectively extracted and enhanced, and the image background noise was significantly weakened. Then, information entropy analysis of the image revealed that compared with the unprocessed original particle image, the reconstructed particle image contained more effective details of the particles with higher information entropy. Based on reconstructed particle images, a more accurate flow field can be obtained within a lower error range.

## 1. Introduction

The flow phenomenon exists widely in various current research fields and is becoming more and more complex and changeable. Examples include vortex flow, jet flow, combustion flow, and multiphase flow. This has led to high requirements for measuring and testing to satisfy the actual requirements in the study of multiform flow fields, from single points to multiple points, single phase to multiphase, planes to space, steady state to transience, and low precision to high precision.

Particle image velocimetry (PIV, for short hereinafter) technology, born in the late 1970s, can meet the abovementioned requirements effectively and is playing an increasingly important role in flow field measurement and visualization, with the advantages of being transient, multipoint, contactless, and highly precise [1]. Data information of spatial structures and flow characteristics can be obtained through recording and analyzing particle information within continuous images via a PIV system containing a light source system, particle image (PIV image, for short hereinafter) acquisition system, control coordination system, tracer particle generating system, special particle image data processing system, and flow field display system [2]. Once the data about the flow field have been obtained, the entire flow field can be visualized. In the analytical data information of the flow field, some inaccurate data may occur, making the flow field inaccurate, especially when the percentage of the inaccurate data is more than 2% [3].

Four types of error exist in particle image velocimetry technology, named random error, acceleration error, velocity gradient error, and following performance error [4]. Among these, random error, also called image background noise, caused by optical noise signals during PIV image recording and analyzing is the most common and contributes the least accurate data. In order to eliminate inaccurate data, three research hotspots, namely particle tracking algorithms, PIV image processing, and PIV array data processing have been studied recently, especially the first of these. Image processing methods mainly include the spatial domain method and frequency domain method. Basically, spatial domain methods including average filtering, median filtering, Gaussian filtering, and bilateral filtering pay great attention to calculating the operation of pixels in the spatial domain, while frequency domain methods focus more on frequency characteristics of the image, especially placing great emphasis on drastic changes of grayscale, or so-called image detail [5]. For PIV image processing, the segmentation of digital plane curves developed by Jose et al. [6] is a valuable technique. The segmented image can describe the target contour in a compact form, which is convenient for higher-level visual processing. Grant et al. [7] summarized early applications of neural networks for PIV image post-processing and analyzed the advantages of this approach in PIV image optimization, image enhancement, and pattern recognition. Shubhra et al. [8] used texture segmentation image processing technology based on gray-level co-occurrence matrices to accurately estimate the PIV two-phase separation interface. In order to reduce noise in image data compression, Dellenback et al. [9] used an automatic movement algorithm based on mass conservation to increase image contrast and minimize the number of unrelated particle images. Shi et al. [10] developed PIV processing software able to automatically select a particle tracking algorithm according to the characteristics of input PIV images, providing a new tool for accurate and efficient processing of PIV images. Wang et al. [11] pointed out that compared with frequency domain methods, the two basic methods in the spatial domain, namely, deleting real information and adding virtual information, definitely add new false interference information to PIV images. Especially when interference information is added to the image boundary, the processing effect of spatial domain methods on that area is very limited. However, in the field of image research, frequency is an indicator of changes in the grayscale intensity of images. The higher the frequency is, the stronger the grayscale changes. Different frequency information plays different roles in the PIV image structure. The main component of the PIV image is the low-frequency image background, while the useful high-frequency particle information occupies a small proportion.

In addition to the aforementioned PIV image processing techniques, another important image processing technique is the wavelet transform. Wavelet transform analysis plays an important role in image processing and is also applied to PIV image processing. Li [12] proposed wavelet-based vector compression technology that could effectively eliminate incorrect PIV vectors, reduce the physical storage, and realize the correction of the error vector by improving the compression ratio. Huo et al. [13] proposed a visual security image encryption scheme based on two-dimensional compressed sensing (2DCS) and integer wavelet transform (IWT) embedding to ensure the security of data. By increasing the speed of the decryption algorithm, IWT embedding can achieve visual security without losing information. For image denoising using wavelet transform, Sun et al. [14] proposed a truncated total variation image denoising model based on a fractional B-spline wavelet. The model effectively suppressed noise and maintained the structure and details of the image. Using wavelet transform image decomposition, Dannina et al. [15] proposed a content-based image retrieval system based on wavelet transform with texture and shape feature fusion, which extracted texture features from images under the premise of wavelet transform and helped to accurately find and retrieve visually similar images from existing databases. The accuracy of that system was higher than that of existing methods.

Information entropy, or Shannon entropy, proposed by Shannon in 1948, is described as a statistical feature of information sources, similar to the concept of entropy in thermodynamics [16]. Shannon was first to conduct research based on information reliability transmission, using mathematical tools such as probability theory and statistics to deduce the calculation method of information entropy. After Shannon, Wiener [17] put forward a statistical mathematical formula of information from the perspective of established filtering theory and signal prediction theory, and the calculation result of information entropy were the same as Shannon’s, thus realizing the quantitative analysis of information. Later, Brillouin [18] provided a satisfactory mathematical proof of information entropy based on the difference between thermodynamic entropy constrained by energy and information entropy not constrained by energy. It can be said that information entropy, as a metric of information, has been strictly mathematically proven. Information entropy solves the global problem of information size and is widely used in the field of information as well as image processing.

In this research, a specific frequency domain method widely used in signal processing, namely wavelet decomposition and reconstruction, was applied to pre-process PIV images in the frequency domain and eliminate the background noise of the PIV images. In order to confirm the effect of wavelet decomposition and reconstruction on the PIV image, the information entropy of the processed PIV image was calculated and analyzed quantitatively. Image quality was judged based on information entropy [19,20].

## 2. Research Method

Wavelet decomposition and reconstruction represent a highly accurate time-frequency domain analysis method, which can be used to study the local characteristics of non-stationary signals. Wavelet decomposition can convert the data of a single image into multi-resolution sub-band areas in a set of independent, spatially oriented frequency channels. It can decompose image signals into high-frequency components and low-frequency components, applying down-sampling according to wavelet basis functions. The low-frequency components of images are extracted in three directions: diagonal (*d*, high–high sub band), horizontal (h, high–low sub band), vertical (v, low–high sub band). Then, wavelet reconstruction can be obtained a high-quality enhanced PIV image through inverse wavelet transform. A more in-depth introduction to wavelet decomposition and reconstruction can be found in the classic textbook *Ten Lectures on Wavelets* [21]. Here, the wavelet decomposition and reconstruction are briefly described as follows. 

For an image, *f* (**t**) ∈ L^2^(R^2^), its 2-D discrete wavelet transform [22] can be expressed as:

(1) $$f(t)=\sum_{k}c_{j_{0},k}\phi_{j_{0},k}(t)+\sum_{j\geq j_{0}}\sum_{k}\sum_{i}{d^{i}}_{j,k}{\psi^{i}}_{j,k}(t)$$

where *j* represents the decomposition level, **t** is a two-dimensional timeline and **t** = (*t*_1_, *t*_2_) ∈ R^2^, I ∈ {*h*, *v*, *d*}, where the symbols *h*, *v*, *d* stand for horizontal, vertical and diagonal directions, respectively. **k** is the location factor, **k** = (*k*_1_, *k*_2_) ∈ Z^2^, and:

(2) $$\phi_{j,k}(t)=2^{j}\phi (2^{j}t_{1}-k_{1},2^{j}t_{2}-k_{2})$$

(3) $${\psi_{j,k}}^{i}(t)=2^{j}\psi^{i}(2^{j}t_{1}-k_{1},2^{j}t_{2}-k_{2})$$

Through the pyramidal methodology calculation, the approximation coefficients *c*_*j*,**k**_ and the detailed coefficients *d*_*j*,**k**_ can be obtained as follows:

(4) $$c_{j,k}=\int_{R}f(t)\;\phi_{j,k}(t)dt$$

and

(5) $${d^{i}}_{j,k}=\int_{R}f(t)\;{\psi^{i}}_{j,k}(t)dt$$

The approximation coefficients represent the rough feature of the original image signal in the low-frequency domain, while the detailed coefficients represent the detail feature of the image signal in the high-frequency domain. Therefore, the original image can be perfectly reconstructed by using these wavelet coefficients [23,24]. Here, the wavelet transform essentially identifies matching between the function f(t) and the mother wavelet ψ(t) [25]. For image processing, the Bior 3.7 wavelet has been proposed to accurately extract the signal characteristics, which has a good effect on PIV images doped with optical noise [26].

The information entropy in the image field (namely image entropy) is a representation of information content and the agglomeration characteristic of grayscale distribution. It can be calculated through the average bit number of grayscale set, in units of bits per pixel (bpp). The computational formulas of information entropy of the image are defined as follows:

(6) $$H_{1}=-\sum_{i=0}^{255}P_{i}\log_{2}P_{i}$$

where *H*_1_ is the one-dimensional information entropy of an image, reflecting aggregation information, and *P_i_* is the proportion of the pixels with gray value *i* in the image. The upper limit of gray scale in an image is 255;

(7) $$H_{2}=-\sum_{i=0}^{255}P_{ij}\log_{2}P_{ij}$$

where *H*_2_ is the two-dimensional information entropy of an image, reflecting both aggregation information and spatial features, and *P_ij_* is the proportion of the array constituted by the gray value *i* of a pixel and gray value *j* of its conterminous pixel in the image. 

Two-dimensional entropy *H*_2_ was selected and Matlab software (https://github.com/YIN-arch/wavelet, accessed on 1 May 2024) was used to calculate the entropy value. Four conterminous pixels are considered in Equation (7) for each pixel situated in the middle of the image, acquiescently forming a 9 × 9 computational domain of a size that can ensure both computational accuracy and computational speed. The higher the information entropy is, more detailed high-frequency information occurs, bringing the image more into focus. The flow field is more accurately obtained by analyzing PIV images with high information entropy, whereas for images with optical noise and background noise, information entropy is relatively low and the obtained flow field is not accurate.

## 3. Results and Discussion

Two typical flow examples in a T-junction channel, separation flow and mixing flow, were used to illustrate the application of wavelet decomposition and reconstruction.

### 3.1. Separation Flow in a T-Junction (Air) [27]

**Figure 1:**
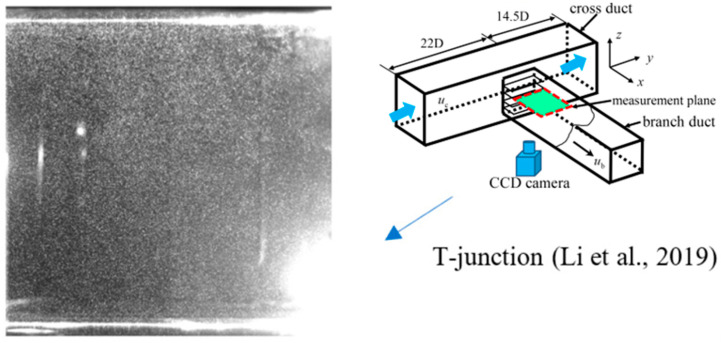
Original PIV image, H_2,o_ = 5.6508 bits/pixel [27].

**Figure 2:**
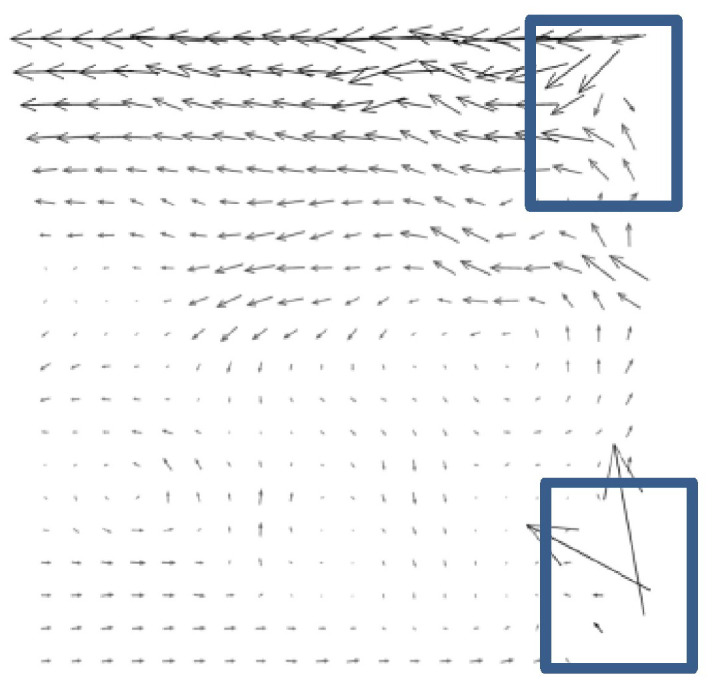
Original analytical flow field of low quality.

A PIV image collected near the branch entrance of a T-junction duct was chosen as the study object. The specific PIV experiment can be found in the published literature [27]. The air was drawn in the cross duct of T-junction and a small quantity of air was separated into the branch duct. The PIV image was captured at the entrance of the branch duct. Figure 1 and Figure 2 show one of original PIV image pair with obvious background noise caused by external optical disturbance, along with its low-quality analytical flow field. The background noise in the original PIV image covered up part of the effective particle information, resulting in comparatively low information entropy *H*_2,o_ of 5.6508 bits/pixel and a low-quality flow field with some distinctly inaccurate velocity vectors, shown in the lower right corner and upper right corner of Figure 2, marked in blue rectangles.

**Figure 3:**
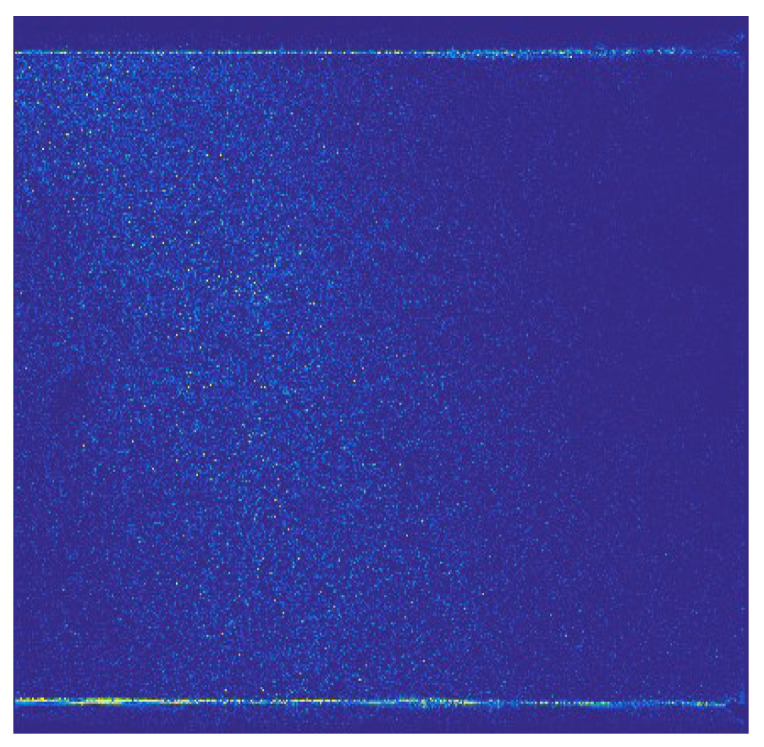
Horizontal details of the original PIV image, H_2,o_*^h^* = 6.0086 bits/pixel.

**Figure 4:**
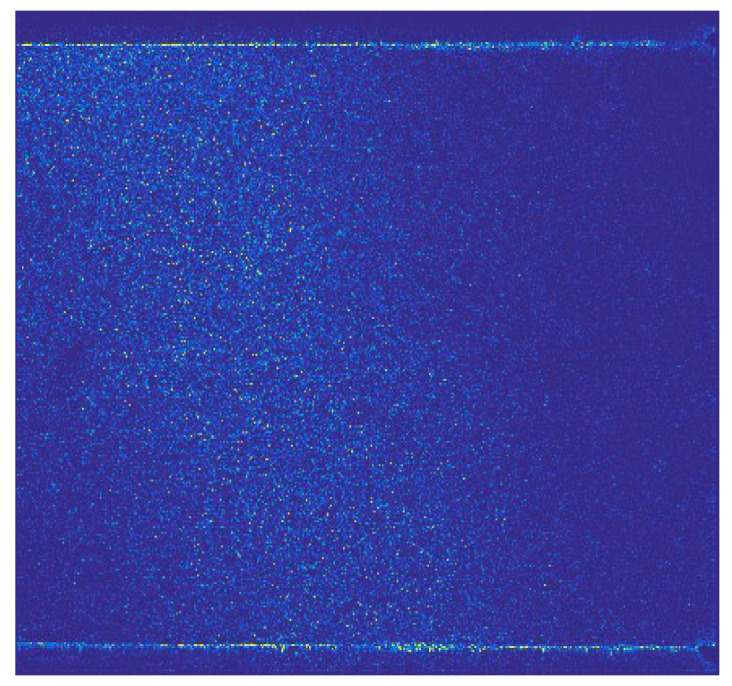
Vertical details of the original PIV image, H_2,o_*^v^* = 6.4471 bits/pixel.

**Figure 5:**
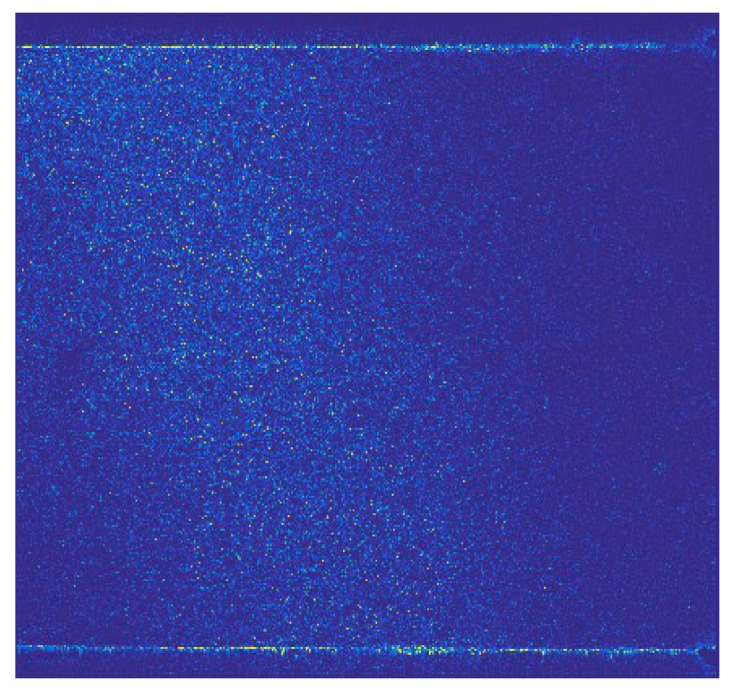
Diagonal details of the original PIV image, H_2,o_*^d^* = 5.7277 bits/pixel.

Wavelet decomposition of PIV images in the frequency domain can extract high-frequency details of the original PIV images along the horizontal, vertical, and diagonal directions. The horizontal details of the original PIV image with information entropy of *H*_2,o_*^h^* = 6.0086 bits/pixel are shown in Figure 3, the vertical details with *H*_2,o_^v^ = 6.4471 bits/pixel are shown in Figure 4, and the diagonal details with *H*_2,o_^d^ = 5.7277 bits/pixel are shown in Figure 5. Among the three figures, it can be observed that the information entropy along the vertical direction is maximal and that along the diagonal direction is minimum. This phenomenon reflects the low-quality of the original PIV image due to the inhomogeneous density of the tracer particles’ distribution and the optical noise, so the high-frequncy PIV image details are also distributed unevenly, leading to inaccurate flow field information. In fact, for high-quality PIV images, the information entropies along the three directions should be the same. 

**Figure 6:**
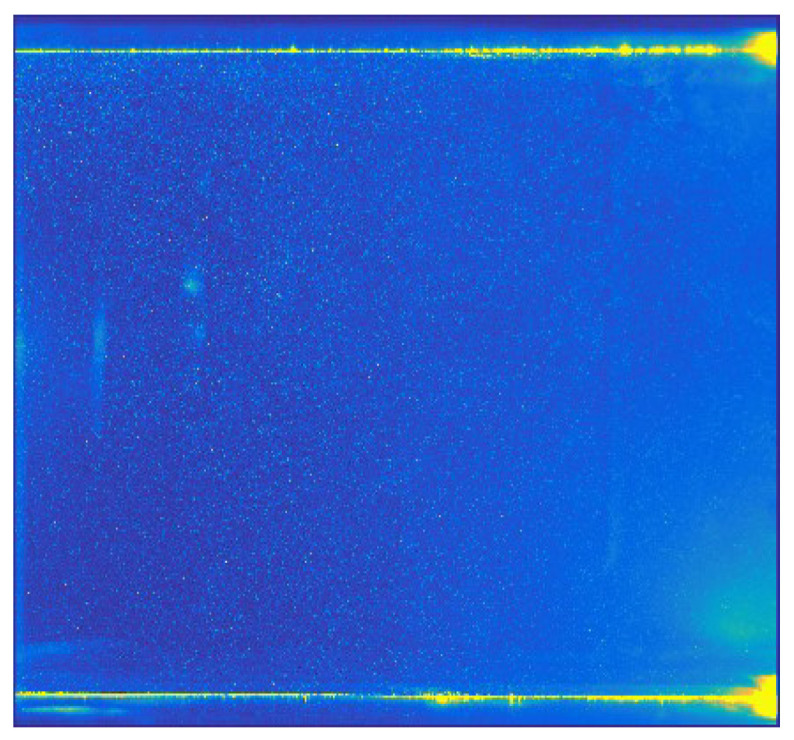
Reconstructed PIV image, H_2,r_ = 7.5301 bits/pixel.

The enhanced high-quality PIV image with information entropy of H_2,r_ = 7.5301 bits/pixel was reconstructed by performing wavelet reconstitution of the extracted details in the abovementioned three typical directions, as shown in Figure 6. The information entropy of each figure during the PIV image processing is listed in Table 1. The information entropy of the reconstructed PIV image was 33.26% higher than that of the original PIV image. In addition, the horizontal details accounted for 79.79% of the reconstructed image and those of the vertical details and diagonal details for 85.62% and 76.06%, respectively. The yellow area in Figure 6 indicates that the details in this area of the original PIV image had not been effectively extracted. This is because not all of the image details in the original PIV image existed along the above three typical directions. Here, three typical directions were selected to extract most of the details in the PIV image, and a small proportion of the details in the PIV image were omitted. If a more accurately reconstructed PIV image is required, the PIV image details can be extracted along more directions, and wavelet transform then performed to reconstruct a more accurate PIV image, obtaining further high-quality information about its flow field. 

**Figure 7:**
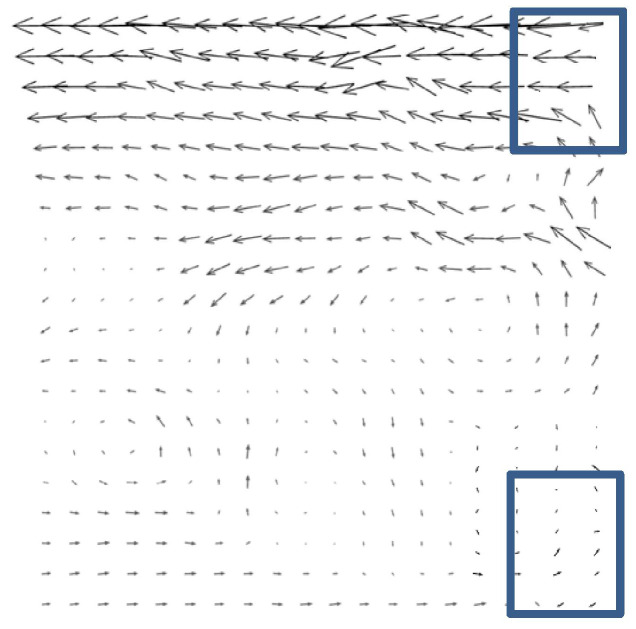
Reconstructed analytical flow field.

Figure 7 depicts a relatively higher-quality analytical flow field obtained from PIV images of enhanced structures with detailed components. The flow field characteristics shown in the reconstructed flow field are consistent with the published research results [27]. It can be clearly seen that the background noise caused by the optical disturbance in the original PIV image has been effectively eliminated, and the processed flow field is more accurate, especially the flow field in the lower right corner and upper right corner of Figure 7 in comparison to Figure 2.

**Figure 8:**
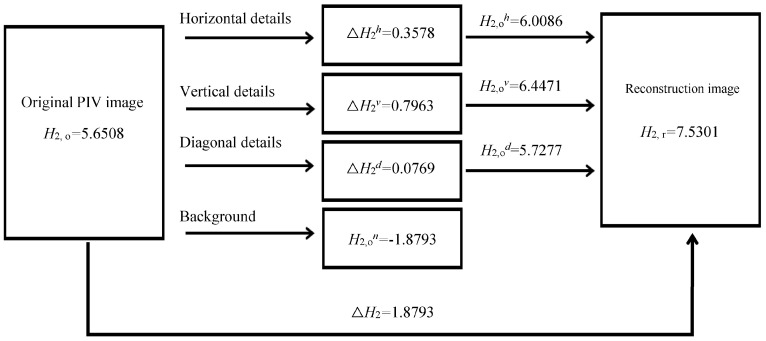
Transitive graph of information entropy during PIV image processing (unit: bit/pixel).

Figure 8 shows the transitive graph of the information entropy during the PIV image process. Regarding the change of information entropy, it was found that in the process of extracting the details of the PIV image, the background of the PIV image was weakened and the image details highlighted, which increased the information entropy. It is worth noting that the sum of three information entropy productions ∆*H*_2_ = (∆H_2_*^h^* + ∆*H*_2_*^v^* + ∆*H*_2_*^d^*) = 1.231 obtained through extracting PIV image details along the three typical directions was less than the whole information entropy production ∆*H*_2_ = (H_2,r_ − H_2,o_) = 1.8793, which means that there were overlaps between PIV image details along the different directions. In addition, the information entropy (H_2,o_) of the PIV image background was negative, indicating that the details of the image background were too few, making the details of the entire PIV image not prominent enough. Therefore, the flow field obtained through PIV image analysis was not accurate.

### 3.2. Mixing Flow in a T-Junction (Water)

**Figure 9:**
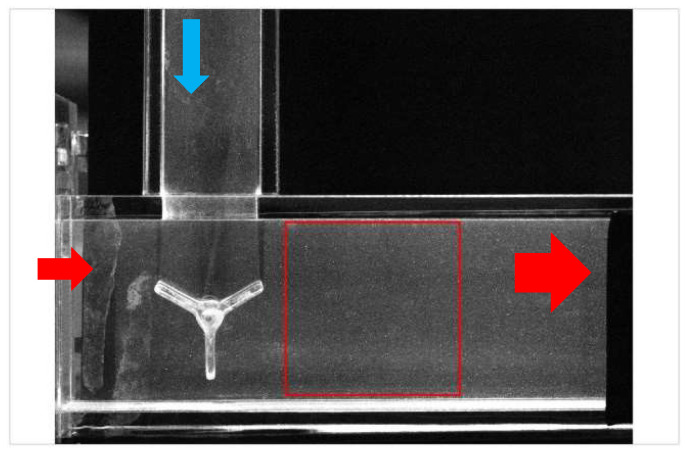
PIV image in mixing T-junction.

An image of the velocity field in T-junction with impellers was used to verify the flexibility of the wavelet method. The working fluid was water. For example, for Case P3-20 (0.5), the maximum velocity was 0.561 m/s. The fluid velocities for the main and branch fluids were 0.1 and 0.2 m/s. The speed of the impeller was 20 rpm. The original PIV image is shown in Figure 9. The area marked in a red square was selected to analyze the information entropy based on wavelet transform. The deviation of information entropy between the first frame and second frame in the original images was 2%. The increases in information entropy between the original and reconstructed images for the first frame and the second frame were 1.5% and 1.6%, respectively, as listed in Table 2.

**Figure 10:**
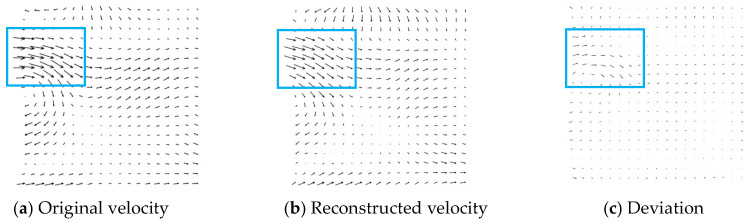
Deviation between the original and reconstructed velocities (*U*_max_ = 0.561 m/s).

**Figure 11:**
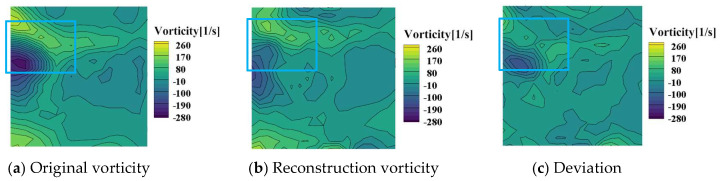
Deviation between the original and reconstructed vorticities (Vor_max_ = −273 s^−1^).

Figure 10a shows the original velocity, and Figure 10b shows the reconstructed velocity. Figure 10c presents the deviation between the original and reconstructed PIV velocity. The maximum deviation was located at the backflow area (blue rectangle). The mean square error of the two velocity was RMS_vel_/*U*_max_ = 9.25%. The vorticity between the original and reconstruction images in Figure 11 was also compared, with a MSE of 5.03%. This result verifies that the information entropy is suitable for evaluating the quality of images and wavelet transform is also feasible for denoising and reconstructing PIV images.

## 4. Conclusions

In this study, wavelet decomposition and reconstruction methods were applied to pre-process a PIV image, and information entropy analysis of the PIV image was used to verify the wavelet processing effect. The main conclusions are drawn as follows: 

Wavelet decomposition and reconstruction is an effective frequency domain method, which can extract PIV image details in different directions and perform image enhancement in the frequency domain to make the reconstructed analytical flow field more accurate. In this study, due to the enhancement of the image details, the information entropy of the reconstructed PIV image was higher than that of the original PIV image. In addition, the PIV image details extracted in different directions partially overlapped each other, so that the sum of the entropy production of the details extracted in different directions was less than the entire production of information entropy. The background information entropy of the low-quality original PIV image was negative, revealing a lack of useful image details and interference with PIV image analysis. 

The information entropy of high-quality PIV image details extracted from different directions should be approximately equal. The larger the difference in the information entropy in different directions, the lower will be the quality of the PIV image. In addition, no matter what PIV image post-processing enhancement method is adopted, the principle of PIV image processing is to highlight the useful detailed information about particles, increase information entropy, and not add new false interference information.

## Figures and Tables

**Table 1:** The information entropy H_2_ of each figure (unit: bits/pixel).

Original PIV Image	Horizontal Details	Vertical Details	Diagonal Details	Reconstruction Image
5.6508	6.0086	6.4471	5.7277	7.5301

**Table 2:** The information entropy of images acquired in a mixing T-junction, based on wavelet transform (unit: bits/pixel).

The first frame PIV image				
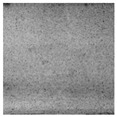	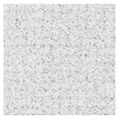	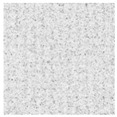	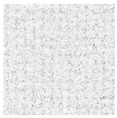	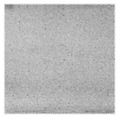
H_2,o_ = 5.9227	H_2,o_*^h^* = 5.6668	H_2,o_^v^ = 5.5780	H_2,o_^d^ = 5.6752	H_2,o_^c^ = 6.0158
The second frame PIV image				
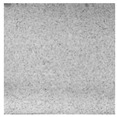	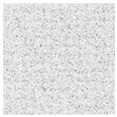	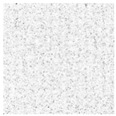	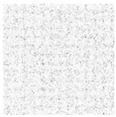	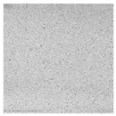
H_2,o_ = 5.7979	H_2,o_^h^ = 5.5974	H_2,o_^v^ = 5.5270	H_2,o_^d^ = 5.7198	H_2,o_^c^ =5.8908

## Data Availability

The raw data supporting the conclusions of this article will be made available by the authors on request.
